# JADPRO Live at APSHO 2016: United in Practice, United in Care

**DOI:** 10.6004/jadpro.2017.8.3.1

**Published:** 2017-04-01

**Authors:** Pamela Hallquist Viale

This past November, over 1,000 nurse practitioners, PAs, pharmacists, clinical nurse specialists, and other oncology professionals gathered at the Gaylord National Hotel in National Harbor, Maryland, to participate in the JADPRO Live at APSHO conference. This meeting was the fourth annual JADPRO Live event addressing the unique educational and professional needs of the advanced practitioner in hematology and oncology. 

The theme of this JADPRO Live, *United in Practice, United in Care*, recognized the importance of all members of the oncology care team. Taking place only a few miles from our nation’s capital, the conference presentations often featured a collaborative educational approach involving two or more speakers discussing practice-changing information. A frequent topic was the role of the advanced practitioner in providing high-quality and effective care. This role was often highlighted in the context of case studies presented by speakers to the audience. 

**Figure 1:**
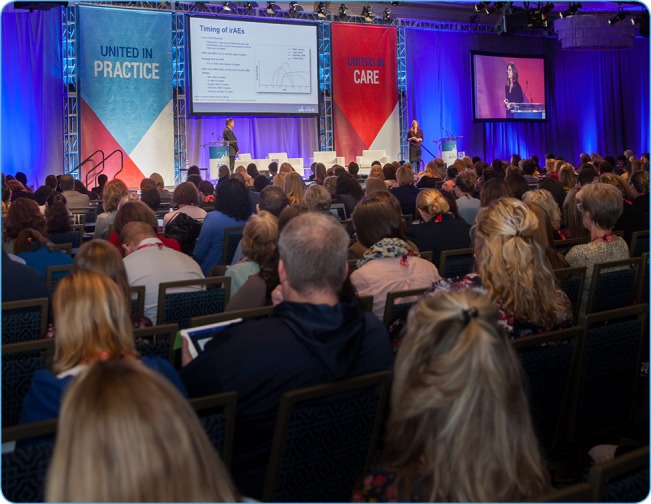


## THE CONFERENCE STORY

The concept for JADPRO Live at APSHO came from a vision for a place where readers of the *Journal of the Advanced Practitioner in Oncology (JADPRO)* could come together and share their expertise with other advanced practitioners. As Conference Chair of the live meeting and Editor-in-Chief of the journal, I’m very proud of the focused education we’ve been able to bring to the advanced practitioner, including sharing clinical updates and practical information, and the opportunities we provide to promote networking among other passionate attendees of the conference.

The JADPRO Live meeting also serves another purpose. It is the setting for the annual meeting for the Advanced Practitioner Society for Hematology and Oncology (APSHO). Along with 4 days of live presentations, members share APSHO society highlights and accomplishments of the past year. In addition, APSHO members who are involved in one of the three APSHO committees (Education, Membership, and Communications) convene to review, discuss, and plan the society’s educational offerings, membership opportunities, and communication strategies. At every meeting, members and attendees are encouraged to network and share important practice updates with each other in both relaxed and more formal settings throughout the conference. 

**Figure 2:**
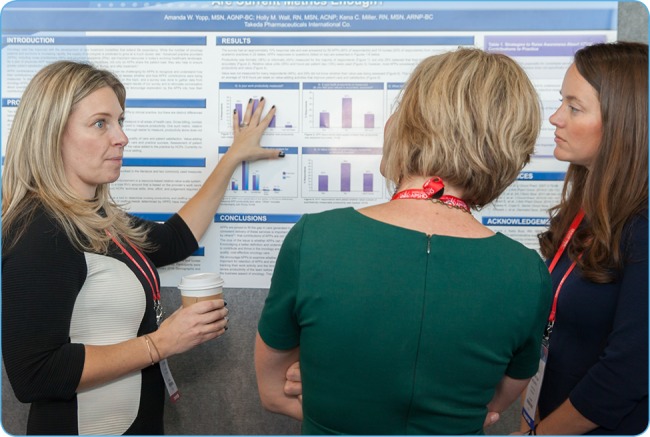


## JOURNAL HIGHLIGHT

To disseminate much of the key meeting information to our readers, we’ve gathered the live meeting content in the print journal. In the pages of this very special issue of JADPRO, you’ll find summaries of the main presentations that were given in National Harbor. 

Our writers have distilled each presentation into its key elements, with the hope of bringing you an overall sense of the meeting. Whether or not you were able to attend the event in person, I think you will find value in this special issue. I hope you will enjoy exploring the critical issues addressing the advanced practitioner in oncology as you read the coverage here. 

## ADDITIONAL EDUCATIONAL OPPORTUNITIES

If you find the following information useful, please visit the JADPRO website (advancedpractitioner.com/sessions) to access full-length presentations recorded at the conference. There you’ll see all of the different learning formats that you can download: full-length video presentations, podcasts, slides, and written transcripts. Choose your preferred format and enjoy the educational offering. Then, follow the instructions to complete a posttest and evaluation and earn your free CE/CME/CEU credits.

**Figure 3:**
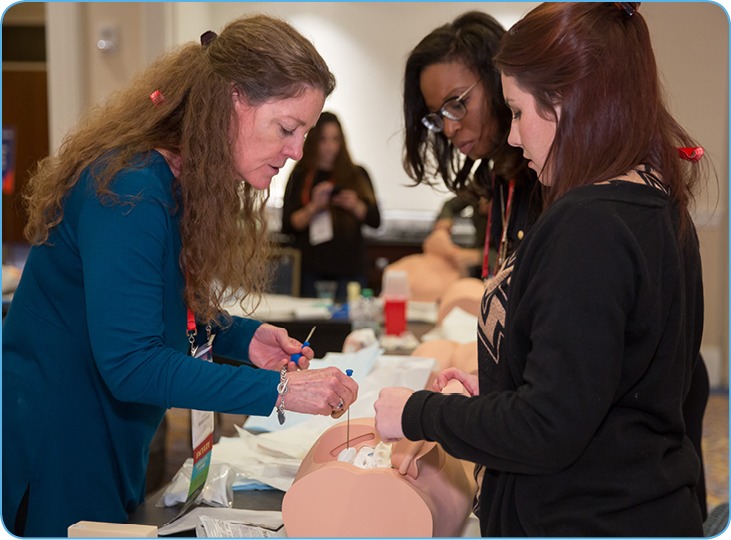


## SEE YOU IN HOUSTON

This special issue of JADPRO gives an overview of the kind of educational content you’ll find at any of the JADPRO Live at APSHO conferences. But what you won’t necessarily see in these pages is the excitement and fun and passion that’s evident as you walk around the conference site, in the main session rooms, the preconference workshops, the exhibit hall, the poster sessions, the luncheons and receptions-—anywhere advanced practitioners gather and network with their peers. That energy comes from the feeling of being recognized, respected, and heard. And that’s what attendees tell us they feel at JADPRO Live. 

Please join us this fall at the Marriott Marquis Hotel in Houston, from November 2 through 5, to participate in a truly unique educational experience and to network with your peers. This year’s theme is *Precision Thinking, Practical Knowledge* and will address many of the exciting updates in oncology care today. Visit jadprolive.com for more information as it becomes available. See you in Houston!

—*Pamela Hallquist Viale, RN, MS, CNS, ANP*

*Chair, JADPRO Live at APSHO*

